# Bilateral Parsonage-Turner Syndrome in a Young Adult Man Due to Parvovirus B19 Infection

**DOI:** 10.7759/cureus.114131

**Published:** 2026-08-07

**Authors:** Alvee Saluja, Akhil Sahib, Samrin Haq

**Affiliations:** 1 Neurology, Lady Hardinge Medical College, New Delhi, IND; 2 Radiodiagnosis, Lady Hardinge Medical College, New Delhi, IND

**Keywords:** bilateral brachial plexitis, human parvovirus b19, pan-brachial plexopathy, parsonage-tuner syndrome, viral trigger

## Abstract

Parsonage-Turner syndrome (PTS) is a rare neuromuscular disorder. Parvovirus B19 infection commonly causes a childhood exanthematous illness. Most previous case reports have documented unilateral parvovirus B19-associated PTS along with the systemic manifestations of parvovirus infection. This report highlights parvovirus B19-associated bilateral PTS without systemic manifestations. A 20-year-old gentleman was asymptomatic one and a half months ago when he developed an acute onset of excruciating right shoulder pain. After 7-10 days, he developed weakness and wasting in the right shoulder girdle. One month into the illness, he developed similar complaints in the left shoulder. At presentation, there was marked wasting of the bilateral deltoids, biceps, triceps, and the periscapular muscles. The motor power was Medical Research Council (MRC) grade 0/5 at the right shoulder, elbow, and wrist joints and grades 1/5 and 4/5 at the left shoulder and elbow joints, respectively. Bilateral hand grip was weak. The deep tendon reflexes were absent in both upper limbs. Touch, pain, and temperature sensation were lost by 50-60% over the right upper limb and by 30-40% over the left upper limb. Nerve conduction studies and electromyography (NCS-EMG) were suggestive of a bilateral pan-brachial plexopathy. The brachial plexus MRI showed short-tau inversion recovery (STIR) hyperintensity involving all the roots, divisions, and cords of the bilateral brachial plexus. Cerebrospinal fluid (CSF) revealed five cells and mildly elevated protein levels. Parvovirus B19 DNA was detected in both the CSF and serum. Serum parvovirus B19 IgG antibodies were positive, while IgM antibodies were negative. Thus, a diagnosis of parvovirus B19-associated bilateral PTS was made. He was given analgesics, gabapentin, nortriptyline, and duloxetine for pain. He received intravenous methylprednisolone (1000 mg) followed by oral steroids (1 mg/kg) along with intravenous immunoglobulin (IVIG) and physiotherapy. Clinicians must consider parvovirus B19-associated PTS as a possibility, especially when evaluating rapidly progressive bilateral brachial plexitis, even in the absence of typical viral prodromal symptoms such as fever, rash, and arthralgia.

## Introduction

Parsonage-Turner syndrome (PTS), also known as idiopathic brachial neuritis or neuralgic amyotrophy, is a rare neuromuscular disorder with an estimated annual incidence of 1.64-3.00 cases per 100,000 persons per year [1,2]. However, it is frequently underdiagnosed, and the actual annual incidence of the disease may be as high as 1 in 1000 persons [3]. PTS classically presents with excruciating unilateral shoulder girdle pain (often worse at night) that lasts for one to two weeks and is followed by profound weakness and wasting of the muscles around the shoulder and arm. The disease commonly affects males in the third to seventh decades of life [4].

The exact etiology of PTS remains unclear. Multiple triggers or risk factors (such as viral illness, recent immunization, trauma, surgery, autoimmune disorders, and drugs) have been associated with the development of the disease. Hence, autoimmune-mediated damage to the brachial plexus triggered by an antecedent event (such as a viral illness), direct viral invasion of the plexus, or ischemia secondary to mechanical trauma or inflammatory etiologies is postulated to be the underlying pathophysiological mechanisms [4,5]. However, an antecedent trigger may not be reported in up to 54% of cases of PTS [6].

Parvovirus B19 (family Parvoviridae) is an icosahedral non-enveloped DNA virus that has a predilection for human erythroid precursor cells. It commonly causes a childhood exanthematous febrile illness with arthralgias, known as "fifth disease" [7]. Parvovirus B19 infections are significantly more common among children than adults, and up to 25% of infections in adults can be asymptomatic [8]. Neurological manifestations affecting the central as well as the peripheral nervous systems have been reported, with encephalopathy, encephalitis, meningoencephalitis, strokes, and peripheral and cranial neuropathies being the most common [9]. Most case series and individual cases of PTS after parvovirus B19 infection have reported unilateral brachial plexus involvement following the systemic symptoms of the infection. Bilateral occurrence of PTS is relatively rare compared to unilateral presentation and occurs in only 10-30% of cases of PTS. We report a rare case of bilateral PTS (that progressed over a few weeks) without any preceding viral symptoms in a young adult male who was found to be infected with parvovirus B19.

## Case presentation

A 20-year-old gentleman born of a non-consanguineous marriage and without any comorbidities was apparently well one and a half months ago, when he developed severe pain in the right shoulder. The pain was worse at night, and the patient described it as 9/10 on the visual analog scale (VAS). The patient did not report any relief in pain despite taking multiple over-the-counter analgesics and described tingling and paresthesias in the right arm and hand a few days after the onset of shoulder pain. After 7-10 days of the onset of pain, he noticed difficulty in picking up heavy objects and placing them on an overhead shelf. He also had difficulty lifting his right shoulder overhead while attempting to put on a shirt. The weakness progressed such that after another one to two weeks, he was unable to lift the right shoulder at all and could not extend the fingers of his right hand. One month after the onset of symptoms, he started having severe pain and paresthesia in the left shoulder. Six weeks into the illness, he could not lift his left shoulder and was unable to extend the left wrist and fingers of the left hand. He did not report any shooting pain from the neck down to the upper limbs, stiffness in the limbs, cranial nerve palsies, band-like sensation over the torso, bladder/bowel complaints, fasciculations, gait imbalance, headaches, seizures, or impaired consciousness. There was no history of significant trauma, fever, rash, joint pain, cough, coryza, or diarrhea before the symptom onset. He did not receive any recent vaccination or intramuscular injection in the arm before the development of his symptoms. He did not have any addictions, and there was no history of chronic herbal medication intake. There was no history of similar episodes and a family history of such limb weakness.

On neurological examination, the higher mental functions were intact. There was no evidence of any cranial nerve palsy. There was a loss of the normal shoulder contour in both shoulders and marked bilateral wasting of the deltoid, biceps, triceps, and periscapular muscles. Hypotonia was noted at the bilateral shoulder, elbow, and wrist joints. The modified Medical Research Council (MRC) grading of motor power was 0/5 at the right shoulder, elbow, and wrist joints in all ranges of motion. The motor power was MRC grade 1/5 at the left shoulder (in all ranges of motion) and 4/5 at the left elbow joint (in flexion and extension). Power at the left wrist in extension and flexion was 0/5 and 3/5, respectively. The small muscles of the hand were weak bilaterally. Bilateral hand grip strength was reduced and was 0% of maximum in the right hand and 50% of maximum in the left hand. The power in the bilateral lower limbs (hip, knee, and ankle joints) was MRC grade 5/5 in all ranges of motion. Touch, pain, and temperature sensation were reduced by 50%-60% over the skin of the right deltoid, right biceps, lateral and medial forearm, and over the right thenar and hypothenar eminences. He reported a reduction of touch and pain sensation by 30-40% over the skin of the left deltoid, left biceps, and the left thenar and hypothenar eminences. The deep tendon reflexes (DTRs) were absent in both the upper limbs and preserved in both the lower limbs. The rest of the neurological examination was normal. 

**Figure 1:**
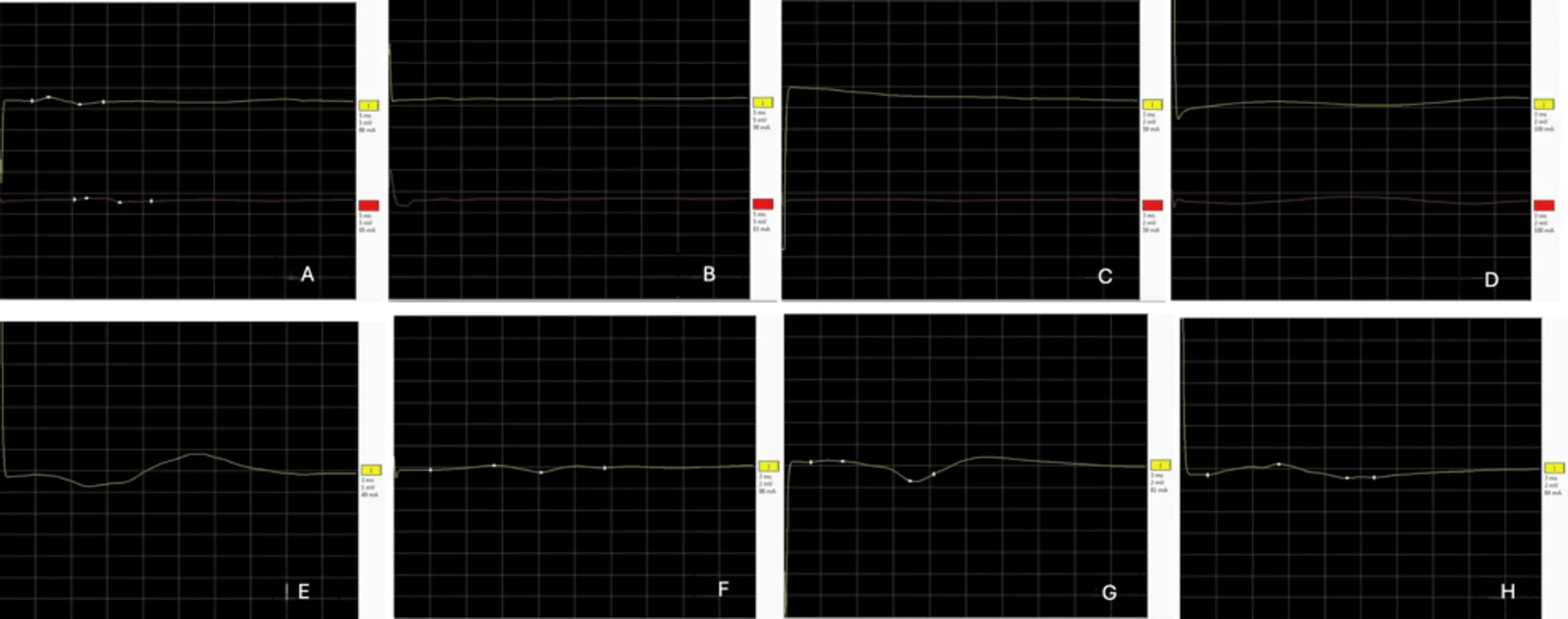
Motor nerve conduction studies in the patient. The figure highlights the motor nerve conduction study graphs that demonstrate markedly reduced CMAP amplitude in the right median nerve (A) and NR response in the left median (B), right radial (C), left radial (D), right axillary (E), and the right musculocutaneous nerve (F). Reduced CMAP amplitude is also noted in the left axillary (G) and the right suprascapular nerve (H). (Sweep speed: 3-5 ms, Gain: 1-5 mV, and current strength range: 60-100 mA). CMAP: compound muscle action potential; NR: not recordable.

His hemogram, liver, renal, and thyroid function tests were within normal limits. Serum human immunodeficiency virus (HIV), hepatitis B surface antigen (HBsAg), and anti-hepatitis C virus (anti-HCV) antibody serology were negative. Fasting blood sugar was 92 mg/dL. Serum vitamin B12 and 25-hydroxy D3 levels were >2100 pg/mL (normal: 180-911 pg/mL) and 23.1 ng/mL (30-100 ng/mL), respectively. Serum angiotensin-converting enzyme (ACE) level was 20.90 U/L (normal: 8-52 U/L). Serum autoimmune nuclear antigen (ANA) antibody, rheumatoid antigen (RA) factor, and extractable nuclear antigen (ENA) profile were negative. Cerebrospinal fluid (CSF) analysis showed five cells (0-4 cells), all lymphocytes, with a CSF sugar of 71 mg/dL (spot dextrose: 116 mg/dL) and a CSF protein of 63 mg/dL (normal: 15-45 mg/dL). A CSF pan-neurotropic viral panel showed parvovirus B19 DNA positivity, and the remaining neurotropic viruses were not detected. Serum parvovirus B19 IgG serology and parvovirus B19 DNA PCR were also positive. The motor nerve conduction studies (NCS) showed reduced or absent compound muscle action potential (CMAP) amplitudes in the bilateral median, axillary, radial, right suprascapular, and right musculocutaneous nerves (Figure 1A-H).

**Figure 2:**
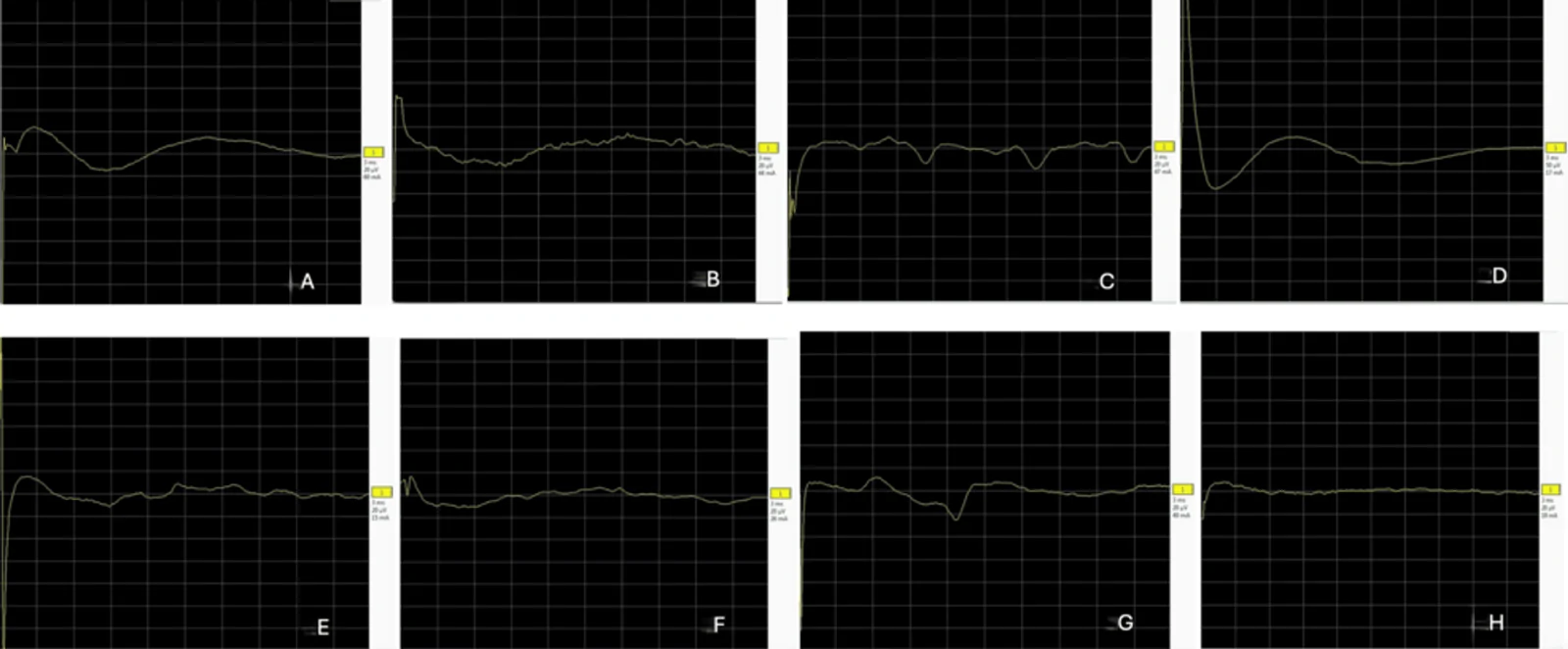
Sensory nerve conduction studies in the patient. The figure highlights the sensory nerve conduction study graphs that demonstrate absent SNAP amplitudes in the right median (A), left median (B), right radial (C), left radial (D), right LABC (E), left LABC (F), right MABC (G), and left MABC (H) nerves. (Sweep speed: 3 ms, Gain: 20-50 mV, current strength range: 15-60 mA). SNAP: sensory nerve action potential; LABC: lateral antebrachial cutaneous; MABC: medial antebrachial cutaneous.

The sensory nerve action potential (SNAP) amplitudes were absent in the bilateral median, radial, lateral antebrachial cutaneous (LABC), and medial antebrachial cutaneous (MABC) nerves. SNAP amplitudes were reduced in the left LABC and left MABC nerves (Figure 2A-H).

**Figure 3:**
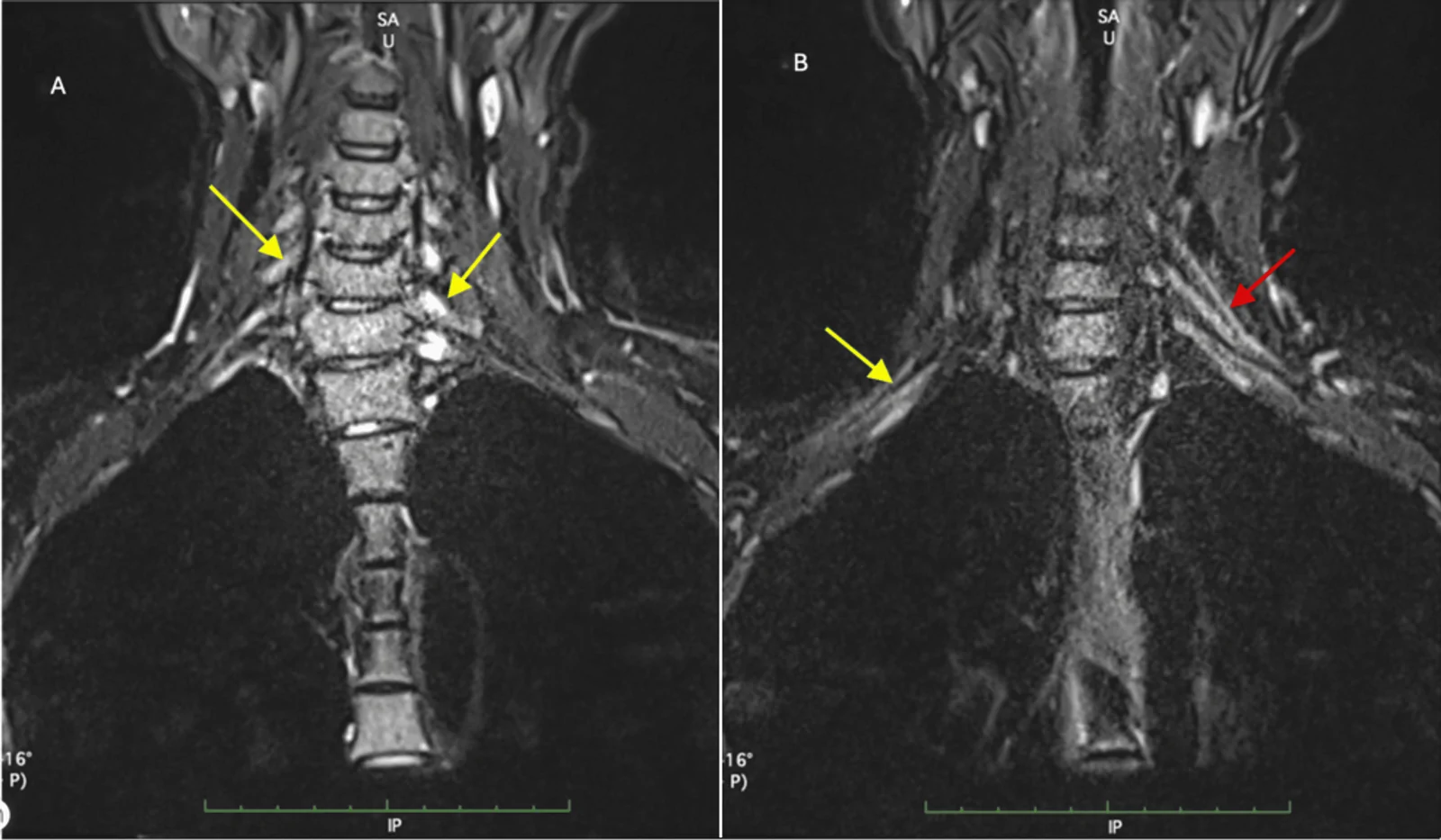
MRI brachial plexus protocol of the patient. (A) A coronal STIR-weighted image showing hyperintense and bulky brachial plexus roots on both sides (yellow arrows). (B) A coronal STIR-weighted image showing hyperintense and bulky right brachial plexus cords (yellow arrow) and bulky brachial plexus roots, trunks, and cords on the left side (red arrow). STIR: short-tau inversion recovery.

Electromyography (EMG) showed fibrillations and positive sharp waves in the bilateral deltoid and triceps, right biceps, right brachioradialis, right abductor pollicis brevis, left extensor indicis proprius, and left extensor digitorum communis. Motor unit action potentials (MUAPs) were not recordable in the bilateral deltoid, right biceps, right triceps, right brachioradialis, left extensor indicis proprius, and left extensor digitorum communis. The MUAPs in the left biceps, left triceps, right abductor pollicis brevis, and right first dorsal interossei muscles were polyphasic, with large amplitude, long duration, and a neurogenic interference pattern. Thus, NCS-EMG were suggestive of bilateral pan-brachial plexopathy. MRI of the brachial plexus revealed bilateral short-tau inversion recovery (STIR) hyperintensity involving all the roots, divisions, and cords of the brachial plexus (from C5-T1) along with marked post-contrast enhancement of the bilateral brachial plexus suggestive of bilateral brachial plexitis (Figure 3A, B).

The relevant investigations of the patient are summarized in Table 1.

**Table 1:** Investigative workup of the patient. ACE: angiotensin-converting enzyme; CSF: cerebrospinal fluid; PCR: polymerase chain reaction; STIR: short-tau inversion recovery; ANA: anti-nuclear antibody; RA: rheumatoid; ENA: extractable nuclear antigen; HIV: human immunodeficiency virus.

S. No.	Parameter (units)	Value	Reference value
1.	Hemoglobin (g/dL)	14.9	13-18
2.	Total leukocyte count (cells/mm^3^)	12,080	4000-11,000
3.	Platelet count (cells/mm^3^)	220,000	150,000-450,000
4.	RBC count (cells/mm^3^)	5.05 million	4.5-6 million
5.	Hematocrit (%)	42.0	40-54
6.	Total bilirubin (mg/dL)	0.33	0.6-1.3
7.	Aspartate aminotransferase (IU/L)	36	5-45
8.	Alanine aminotransferase (IU/L)	32	5-35
9.	Alkaline phosphatase (IU/L)	66	48-128
10.	Serum albumin (g/dL)	4.6	3.5-5.2
11.	Sodium (mEq/L)	138	136-145
12.	Potassium (mEq/L)	3.9	3.5-5.1
13.	Total calcium (mEq/L)	9.6	8.6-10.2
14.	Urea (mg/dL)	22.1	15-40
15.	Creatinine (mg/dL)	0.92	0.6-1.3
16.	Fasting blood sugar (mg/dL)	92	70-100
17.	Thyroid-stimulating hormone (micro IU/mL)	2.56	0.34-5.6
18.	Vitamin B12 (pg/mL)	>2100	180-911
19.	Vitamin D3 (ng/mL)	23.1	30-100
20.	HIV serology	Negative	Negative
21.	Hepatitis B surface antigen serology	Negative	Negative
22.	Anti-hepatitis C antibody serology	Negative	Negative
23.	Creatinine kinase (IU/L)	274	<171
24.	ANA	Negative	Negative
25.	RA factor	Negative	Negative
26.	ENA profile		Negative
27.	Serum ACE levels	20.9	8-52 IU/L
28.	CSF total leukocyte count (cells/mm^3^)	5	0-4
29	CSF sugar (mg/dL)	71	50-80
30	CSF protein (mg/dL)	63	15-45
31	CSF parvovirus B19 DNA (PCR)	Positive	Negative
32	Serum parvovirus B19 serology	IgG positive; IgM negative	Negative
33.	Serum parvovirus B19 DNA	Positive	Negative
34.	MRI brachial plexus protocol	STIR hyperintensity in the bilateral roots, trunks, and cords of the brachial plexus from C5-T1	-

**Figure 4:**
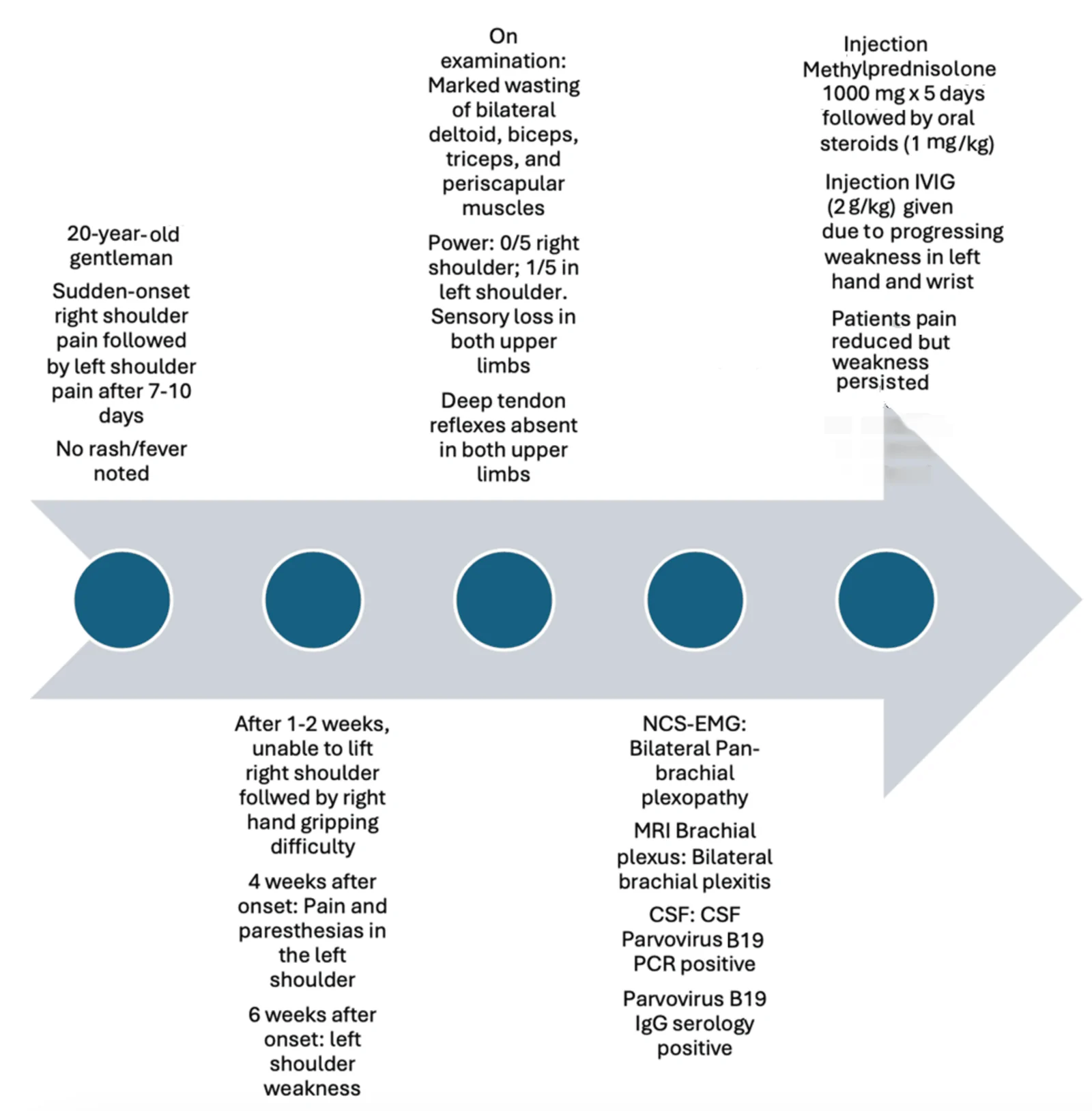
Chronological summary of the case presentation. This figure summarizes the timeline of symptom development along with the examination, investigations, and treatment protocol in this case. NCS-EMG: nerve conduction studies-electromyography; MRI: magnetic resonance imaging; CSF: cerebrospinal fluid; PCR: polymerase chain reaction; IgG: immunoglobulin G; IVIG: intravenous immunoglobulin G.

Thus, based on the history, clinical examination, and investigations, a diagnosis of parvovirus B19-associated bilateral pan-brachial plexitis was established. The timeline of symptom development, along with the examination, investigations, and treatment protocol in this case, is summarized in Figure 4.

Due to excruciating bilateral pain and severe weakness, he was started on injectable pulse methylprednisolone 1000 mg IV once daily × 5 days, followed by oral steroids at 1 mg/kg/day (total 70 mg) for two weeks, with a weekly taper of 10 mg. The patient received oral steroids for a total of six weeks. Along with steroids, the patient also received NSAIDs, gabapentin+nortriptyline (400/10 mg) (1 tablet HS), and duloxetine 30 mg bid for neuralgic pain and paresthesias in the upper limbs. He was advised to perform physiotherapy exercises and to take oral calcium and vitamin D3 supplementation. His pain and paresthesias markedly improved during the hospital stay. After one week of hospital stay, the patient was pain-free but had mild progression of weakness in the left hand and wrist. Due to progressive weakness and parvovirus B19 DNA positivity, he was also administered intravenous immunoglobulin (IVIG) (2 g/kg over five days; total 110 g). At two months of follow-up, the patient is pain-free, and his grip strength has improved to 25% of maximum in the right hand and to 75% of maximum in the left hand. However, his bilateral shoulder weakness persists and has not improved. He remains on close follow-up and has been advised to continue physiotherapy exercises.

## Discussion

We present a rare case of rapidly progressive bilateral brachial plexitis in a young adult male who was subsequently found to be infected with parvovirus B19. Our case presented with acute asymmetric onset (right followed by left) shoulder pain that was subsequently followed by severe weakness of the upper limbs (especially the shoulder girdle). Seventy per cent of cases with PTS present with unilateral, asymmetric, severe shoulder pain that is often described to have a throbbing or burning quality [10]. Apart from the shoulder, patients can report pain in the scapular blade, superolateral thoracic wall, the antecubital fossa, and the lateral forearm. The pain is often worse at night and not relieved by postural change [11,12]. Bilateral PTS is uncommon, occurring in approximately 10-30% of cases [13]. Several conditions can present with unilateral or bilateral shoulder girdle pain and/or shoulder weakness and can thus mimic PTS. Table 2 highlights the differential diagnoses to consider in PTS.

**Table 2:** Differential diagnoses of PTS. PTS: Parsonage-Turner syndrome; NCS: nerve conduction studies; EMG: electromyography; MRI: magnetic resonance imaging; SNAP: sensory nerve action potential; T2: transverse relaxation time.

S.No.	Differential diagnosis	Distinguishing feature from PTS
1.	Cervical radiculopathy	More common in advanced age groups, with neck pain and stiffness, radicular pain and weakness along the affected root, loss of reflexes corresponding to the root involved, SNAPs normal on NCS. MRI of the cervical spine shows a prolapsed disc and root compression.
2.	Mononeuritis multiplex	Asymmetric, sudden-onset weakness, wasting, and sensory loss affecting the distribution of multiple non-contiguous nerves. Sequential involvement of individual nerves is common. Underlying systemic diseases (connective tissue disease, diabetes, vasculitis, leprosy, etc.) can be present. NCS confirms the diagnosis.
3.	Transverse myelitis (cervical)	Lower limb weakness, upper motor neuron signs such as hyperreflexia and spasticity develop during the course of the disease, bladder and/or bowel involvement, a well-defined sensory level or suspended sensory loss. NCS shows normal SNAPs. MRI spine may show T2 hyperintense signal in the cord.
4.	Rotator cuff tear	History of trauma, severe local shoulder pain especially on sleeping on that side, pain on shoulder movement (“clicking” or “popping” on attempting to lift the arm), decreased range of motion of the shoulder joint, no sensory loss, and preserved reflexes. NCS is normal.
5.	Acute calcific tendinitis at the shoulder	Painful movement at the shoulder joint, restricted range of motion at the shoulder joint (especially overhead and to the side), no sensory loss, and preserved reflexes. NCS is normal.
6.	Adhesive capsulitis of the shoulder joint	Passive movement of shoulder is painful and restricted, no sensory loss, reflexes usually preserved, and NCS is normal.
7.	Amyotrophic lateral sclerosis (Man-in-Barrel syndrome)	Insidious onset and gradually progressive painless pure motor disease (no sensory loss). Fasciculations can be seen, SNAPs are spared on NCS, and EMG of paraspinal muscles is abnormal in contrast to brachial plexitis.
8.	Entrapment neuropathy	Pain and paresthesia are in a single nerve distribution, upper limb reflexes are usually spared, specific provocative tests can replicate symptoms, and NCS confirms focal demyelination in the distribution of the nerve.
9.	Multifocal motor neuropathy	Slower progression, painless pure motor disease (no sensory loss), fasciculations can be seen, weakness and wasting in a single nerve distribution, SNAPs are spared on NCS, and conduction blocks noted in motor NCS.
10	Superior sulcus tumor of the lung (Pancoast tumor)	Lower brachial plexus predominantly affected (C8-T1 distribution), systemic symptoms (loss of weight, appetite loss, cough, hemoptysis), and NCS-EMG demonstrates lower plexus affection.
11.	Brachial plexus tumors	Slowly progressive, palpable neck/axillary swelling, and persistent dull pain.

In a cohort of 81 cases with parvovirus B19-associated neurological manifestations, 62 (77%) had CNS manifestations and 19 (23%) had peripheral nervous system involvement. Of those with peripheral nervous system involvement, eight patients had brachial plexitis. Seven of these eight patients (88%) had a rash, five (63%) reported arthralgia, and three (38%) had bilateral involvement. All eight reported patients with parvovirus B19-associated brachial plexitis were adults, and seven of these eight were immunocompetent [14]. However, MRI confirmation of brachial plexitis was not done, and data on the CSF or serum confirmation of parvovirus B19 DNA among the cases with Brachial plexitis were not provided. 

In a systematic review of 129 patients with parvovirus B19 infection-associated neurological manifestations, 41 had peripheral nervous system involvement, with eight patients (19.5%) having symptoms suggestive of neuralgic amyotrophy. In this review, six of the eight patients with brachial plexitis (75%) had systemic symptoms, such as fever, anemia, and arthralgia, that preceded or occurred concurrently with the neurological manifestations. Only two cases of bilateral brachial plexitis were reported in this review [9]. However, neuroimaging was not done among those with brachial plexitis. Furthermore, serum and/or CSF DNA PCR confirmation was not done in most of these cases. Our case presented with bilateral brachial plexus involvement in the absence of the typical viral systemic manifestations (fever, rash, and arthralgias). Table 3 highlights the differences between our case and those reported in the literature.

**Table 3:** Comparison of this case with available literature on parvovirus B19-associated PTS. PTS: Parsonage-Turner syndrome; MRI: magnetic resonance imaging; CSF: cerebrospinal fluid; IgM: immunoglobulin M; IgG: immunoglobulin G.

S.No.	Parameter	Douvoyiannis et al. [14]	Barah et al. [9]	Present case
1.	Type of literature	Retrospective cohort study	Systematic review	Case report
2.	Number of patients with PTS and parvovirus B19	8	8	1
3.	Number of patients with unilateral presentation	5	6	None
4.	Number of patients with bilateral presentation	3	2 (1 patient had systemic symptoms and 1 did not)	1
5.	Percentage of patients with fever	88% (7/8)	75% (6/8 systemic symptoms)	0% (no systemic symptoms)
6.	Percentage of patients with rash	88% (7/8)	75%(6/8 systemic symptoms)	0% (no systemic symptoms)
7.	Percentage of patients with arthralgia	63% (5/8)	75%(6/8 systemic symptoms)	0% (no systemic symptoms)
8.	MRI confirmation of plexitis	Not done/not reported	Not done/not reported	Done
9.	Parvovirus B19 serology (IgM and IgG) testing in plexitis patients	Done	Done	Done
10.	CSF parvovirus B19 DNA testing in plexitis patients	Not reported	Not available or not done	Done (positive)
11.	Serum parvovirus B19 DNA testing in plexitis patients	Not reported	Not done in 5/8 patient. Done in 3 patients (Present in 2 patients, absent in 1 patient)	Done (Present)

In our case, parvovirus B19 serology showed an IgG positivity with an absent IgM response, suggestive of past infection. Since our patient presented 1.5 months after symptom onset, the IgM antibodies had likely declined by the time he was tested (as >60 days had elapsed since symptom onset at the time of testing), while the IgG antibodies persisted [15]. The neurological manifestations of parvovirus B19 can result from multiple mechanisms, including direct neurotropic viral invasion, endothelial damage, immune-mediated vasculitis, autoimmune mechanisms via molecular mimicry, and immune dysregulation [16,17]. The presence of elevated systemic IgG antibodies against parvovirus B19, mildly elevated CSF cells and proteins, and the presence of viral DNA in CSF in our case suggest the possibility of a virus-triggered immune-mediated inflammation and damage to the brachial plexus (VP1 and NS1 viral proteins may mimic myelin basic protein in peripheral nerves, triggering cell-mediated and humoral immunity). However, a direct neurotropic effect of the virus cannot be completely ruled out [18]. Asymptomatic infection with parvovirus B19 can occur in up to 25% of healthy adults [8]. Our case could have had an initial asymptomatic infection before developing the neurological symptoms, which then manifested and progressed once the titers of the systemic antibodies started rising.

The mainstay of treatment for PTS includes analgesics (NSAIDs and/or opioid analgesics), neuroleptics (for neuropathic pain), and physical rehabilitation [4,10,11]. In parvovirus B19-associated PTS, corticosteroids and/or IVIG have been shown to improve neurological symptoms and overall outcomes [9,14]. IVIG may play a particularly beneficial role in parvovirus B19-associated neurological disease by neutralizing the virus [16]. However, long-term physical rehabilitation is required in the chronic phase, and recovery from parvovirus B19-associated PTS can be quite prolonged, ranging from two to six months and sometimes even up to three years [19]. In the PTS cases that fail to improve despite rehabilitation and medical treatment, surgical options include neurolysis and nerve and tendon transfers [20]. 

## Conclusions

We acknowledge the fact that this is only a single case report. We thoroughly investigated all possible etiologies (including rare viral etiologies such as parvovirus B19) given the rapidly progressive bilateral pattern of PTS in our patient. Thus, clinicians must consider parvovirus B19-associated PTS as a possibility, especially when progressive bilateral brachial plexitis is present, even in the absence of typical viral prodromal symptoms such as fever, rash, and arthralgia. Hence, greater clinical suspicion and awareness regarding this presentation may prompt active testing for parvovirus B19. This would provide a more accurate estimate of the incidence of the neurological complications associated with this virus. Furthermore, the role of IVIG alone or in combination with steroids has been shown to improve outcomes in parvovirus B19-associated neurological manifestations. Thus, identifying the underlying etiology may also affect treatment decisions and overall patient outcomes in such cases.
